# Parent-mediated early intervention in infants and toddlers at elevated likelihood for autism: a systematic review of randomized controlled trials

**DOI:** 10.3389/fpsyt.2026.1676968

**Published:** 2026-04-13

**Authors:** E. Conti, F. Ieri, S. Calderoni, F. Apicella, N. Chericoni, V. Costanzo, Viviana Marchi, A. Guzzetta, C. Colombi

**Affiliations:** 1 University of Pisa, Department of Clinical and Experimental Medicine, Pisa, Italy; 2 IRCCS Stella Maris Foundation, Pisa, Italy

**Keywords:** autism, early intervention, evidence-based practice, parent-mediated, preemptive, randomized controlled trial (RCT), toddler outcomes

## Abstract

The current prevalence of Autism Spectrum Disorder (ASD) has risen to 1 in 31, according to a recent report of the USA Centers for Disease Control and Prevention. While prodromal signs of ASD can be observed during the first months of life, most care approaches usually require a diagnosis before children can receive autism-specialized intervention services. A novel approach consists in providing parent-mediated intervention to infants higher likelihood for autism with the aim to decrease disability and perhaps impacting on developmental trajectory. The aim of this review is to summarize evidence on outcomes from Randomized Control Trials (RCTs) of parent-mediated early interventions in infants with very early ASD signs and/or with an elevated likelihood for ASD, in order to inform clinical practice. A systematic literature search was performed by using the following databases from 2014 until 17 February 2025: Pubmed, EMBASE, Scopus, Web of Science, OVID (PsycInfo). Papers were selected based on the following inclusion criteria: i) RCT studies; ii) Mean age of children enrolled in RCT studies ≤ 18 months; iii) English language published studies; iv) Infants presenting autistic signs or infants at elevated likelihood for ASD. Eleven studies were included and analyzed in regard to sample characteristics, enrollment strategies, outcome measures and intervention types.

## 1 Introduction

Autism Spectrum Disorder (ASD) is a heterogeneous condition characterized by atypicalities in social communication and interaction and the presence of restricted interests and repetitive behaviors (1). A recent analysis published in the CDC’s Morbidity and Mortality Weekly Report indicates that 1 in 31 8-year-old children are autistic (2). Such a high incidence brings important consequences, impacting families, society, as well as educational and health systems. Although research suggests that ASD prodromes emerge during the first year of life (2) and early intervention is crucial for improving outcomes (3), children commonly receive a diagnosis beyond 3 years of age and start intervention even later, thereby missing the window of highest brain plasticity (4, 5). Indeed, growing evidence report the possibility to support early development by delivering parent-mediated intervention strategies, in order to increase access to intervention as early as possible for young children at high likelihood of receiving a diagnosis of ASD. Brian et al. (6) in their large community implemented study, showed that an evidenced-based parent-mediated intervention, the Social ABCs, for toddlers with ASD (age range: 14–34 months) can be delivered within community services. Indeed, parents learned the intervention at fidelity level and toddlers made clinically meaningful gains, suggesting that this approach is feasible and effective and may be proposed to families immediately in response to first signs of ASD.

ASD prodromes may be detected in multiple areas of neurodevelopment in the first months of life, including 1) attention, 2) development of communication and prelinguistic communication, 3) affect, temperament and social engagement, 4) sensory sensitivity and habituation, 5) motor abilities, toy play and restrictive and repetitive behaviors (2). Moreover, studies have shown that infants with high ASD likelihood exhibit atypical developmental trajectories either in brain structure or function from the first months of life (7–11). In this framework, specific brain alterations have been linked to particular ASD-related behaviors. For instance, subcortical brain volumes at 4–6 months (12), callosal pathway integrity at 6 months (13), and EEG connectivity at 14 months (14, 15) have all been associated with repetitive behaviors later in development. In a similar vein, early disruptions of functional connectivity in auditory regions (16) and atypical lateralization and ERP responses to auditory stimuli at 6–12 months (17) have been correlated to later language outcome.

Identifying early behavioral and neurobiological markers of ASD is essential for supporting early clinical detection and subsequently implementing early and preemptive interventions. Indeed, a growing body of evidence suggests that providing intervention for ASD before 24 months of age may be especially effective, due to the heightened neurodevelopmental plasticity typical of this early developmental period (18, 19). The study by Lombardo and colleagues (20) supports this notion by demonstrating that children who began intervention prior to their second birthday show significantly greater developmental gains, particularly in language, adaptive behavior, and cognitive functioning, compared to those who started later. These differences underscore the critical role of timing in shaping early developmental trajectories. Guthrie et al. (21) showed that children starting intervention at 18 months of age showed greater gains in receptive/expressive language, social communication, and daily living skills in comparison to children beginning the same intervention at 27 months of age.

Following the principles of brain plasticity and the impact of very early intervention on developmental trajectories, the implementation of pre-emptive parent-mediated intervention during the first phases of life has been proposed with the aim of improving developmental outcomes (22–24). Parent-mediated interventions can help families implement evidence-based strategies to support optimal development within parent-child play and caring activities. It has been observed that this type of early intervention reduces family stress, autistic symptoms and supports developmental skills (25, 26).

Different methodologies of parent mediated intervention have been set up mainly based on developmental frame, such as ART (adaptive responsive teaching), iBAsis VIPP, Baby Jasper (Parent mediated Joint Attention Symbolic Plat engagement and regulation, PFR (promoting first relationship, imPACT (improving parents ad Communications Teacher). They are all centered on the figure of parent in order to promote child development.

These methodologies have been investigated by Hampton and colleagues (2022) (27) in a systematic review and metanalysis reporting either RCT studies or feasibility studies. Results indicated that early intervention was associated with parental implementation of intervention strategies immediately following the intervention but not with positive child developmental outcomes. In a more recent review limited to RCT studies in children below 24 months of age, the authors confirm no clear impact of early intervention on neurodevelopmental trajectories even though a positive trend has been found (28).

However, on the basis of the potential of very early intervention programs during periods of maximum brain plasticity (29) we chose to narrow the age range to under 18 months in order to specifically investigate very early pre-emptive intervention studies. Hence, we systematically reviewed available randomized control trials (RCTs) intervention studies in infants and toddlers at higher likelihood for autism (e.g. siblings, preterm infants) or presenting early ASD prodromes (mean age below 18 months). The objective is to highlight the weaknesses and strengths of these interventions, as well as to critically interpret the results, within the framework of personalized medicine, in order to inform health policies and possibly set-up devoted early intervention services which can have a positive cascading effect on long term development (30).

## 2 Materials and methods

The preferred reporting Items for Systematic Reviews and Meta-Analysis (PRISMA) (31) has been used for this review.

### 2.1 Search strategy

A systematic literature search was performed by using the following databases from 2014 until 17 February 2025: Pubmed, EMBASE, Scopus, Web of Science, OVID (PsycInfo).

The search terms included: “randomized controlled trial” OR “RCT”, AND “Autism Spectrum Disorder” OR “Autism OR early autism”, AND “infant OR toddler” OR “baby” OR “Early childhood”, AND “early therapy” OR “early treatment” OR “early intervention”

References of all included studies and relevant systematic reviews were examined.

### 2.2 Inclusion criteria

Studies that met the following selection criteria were included:

- RCT studies
- Mean age of children enrolled in RCT studies ≤ 18 months
- English language published studies
- Children presenting autistic signs and/or at elevated likelihood for autism

### 2.3 Data extraction

Data were extracted by two authors (E.C., F.I.). The following information has been extracted from each included study: 1) Sample characteristics, sample size, M/F ratio, age at enrollment); 2) Aim of the studies; 3) Type of referral; 4) Inclusion/exclusion criteria; 5) Characteristics of participants at baseline; 6) Type of outcome measures; 7) Type of intervention, duration, method of delivery.

**Figure 1:**
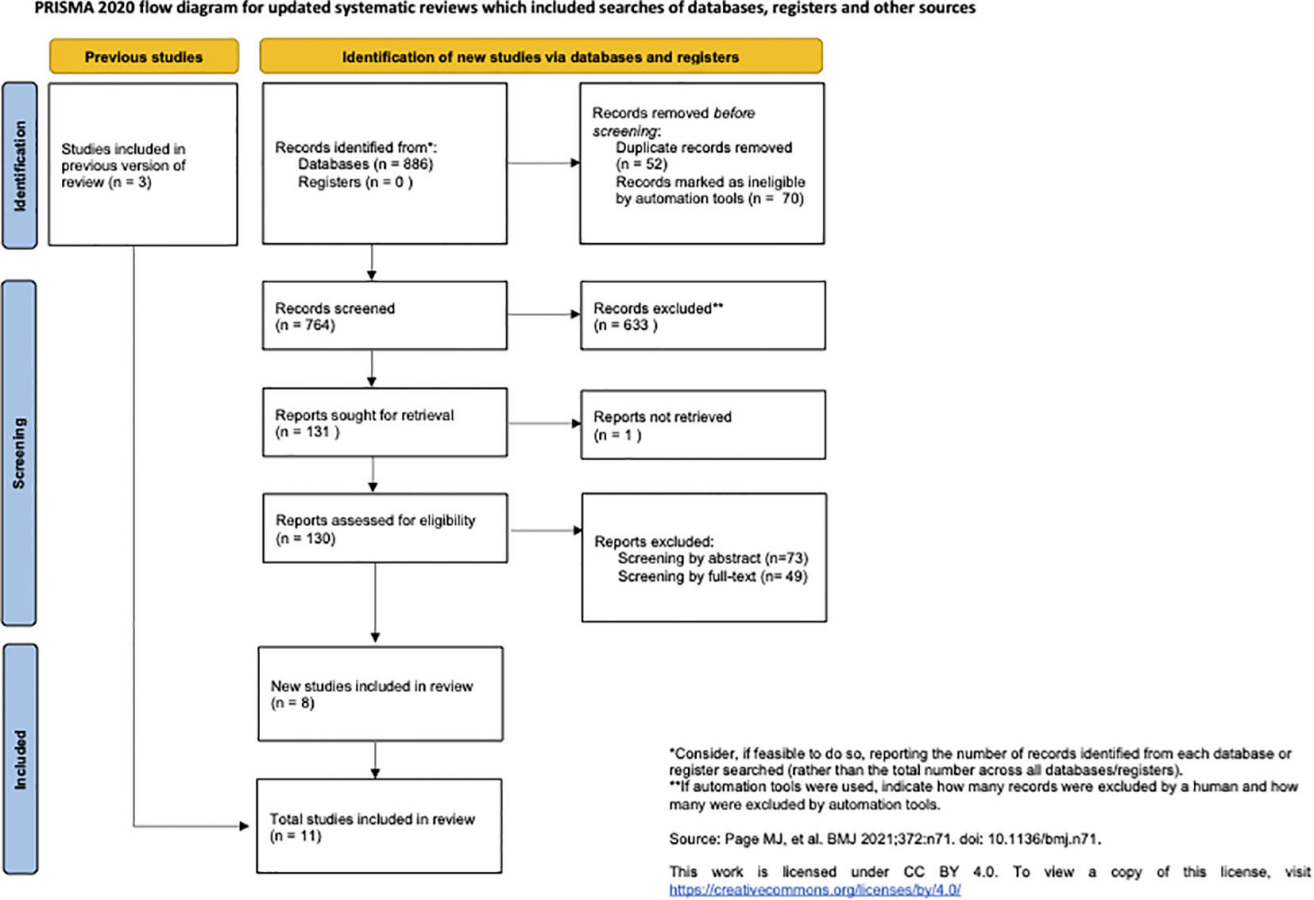
PRISMA. Preferred reporting items for systematic reviews and meta-analysis.

A total of 886 articles were identified from our searches (251 articles from OVID database; 30 articles from EMBASE database, 91 articles from Web of Science, 310 articles from Scopus database; 93 articles from Cochraine Database, 111 articles from Pubmed) and screened by title and abstract by two authors independently (E.C. and F.I.) who excluded 756 papers based on inclusion criteria and duplication. The remaining 130 articles were retrieved for full text and reviewed leading to 8 studies meeting full inclusion criteria (3, 26, 32–37). Three more papers (36, 38) were retrieved from other strategies such as reading references of previously identified studies and all met full inclusion criteria (see PRISMA diagram, **Figure 1**).

### 2.4 Characteristics of included studies

#### 2.4.1 Sample characteristics

The sample size ranges from 16 children (38) to 103 children (3, 26). The age of the participants at enrollment ranges from a minimum of 6 months (34) to a maximum of 22 months (37), with the average median age below 18 months, according to the inclusion criteria. The male-to-female ratio was not available for two studies (3, 40), while the remaining reported a ratio of approximately 3:1 or 4:1, in line with previous data on this topic (41) See **Table 1** for details.

**Table 1:** Characteristics of included studies.

Study	Sample characteristics	Aim of the study	Referral	Inclusion/ Exclusion criteria	Characteristics of participants at baseline	Intervention type	Outcome measures	Significant results
Baranek et al, 2015 (38)	Sample Size: N=16 Age at enrollment: 12 months (Mean age: ≈15) M:F= 14:2	Feasibility and efficacy study	FYI at 12 months by mail Selection: -FYI ≥95°pc -FYI≥90°pc + parental concerns -Family history of ASD	Inclusion criteria: -AOSI ≥ 7, and/or ADOS-T ≥12) and/or -showed delays in 2/3 domains of social- communication [(MSEL (*T* < 40), CSBS (SS < 7), MCDI (<15%), and ITSEA (rated “of concern”)] and/or -showed disruptions in 2/3 domains of sensory-regulatory functions SPA>(1.5.s.d above norm) SEQ(>1.5s.d. above norm) and ITSEA “rated of concern” Exclusion criteria: -Hearing or vision impairments -Down syndrome -Cerebral Palsy -Significant prematurity -<2500 g at birth	Time 0 (around 15 months): -AOSI -ADOS -MSEL -VABS II -CSBS -MCDI -ITSEA -SPA -SEQ -MBRS	**ART intervention group (n=11)**: relationship-focused, home-based intervention **REIM intervention group (n=5):** control group, that received Early Intervention services available in the community, based on parental choice and accessibility Duration: 6 months	Time 1 (22-26 months): -MSEL -CSBS -SPA -SEQ -MBRS -VABS II Time 2 (30-35 months): -MSEL -SPA -SEQ -*A Parent Diagnostic Interview* -MBRS -VABS II -ADOS	-Feasibility of using the FYI to refer to the study -Earlier diagnosis -Both groups received community services earlier -ART showed more beneficial effects relative to REIM (group received EI services) -Significant improvements in receptive language, socialization, and sensory hypo responsivity, alongside less directive parenting style compared to the REIM group, particularly during the 6–8-month active phase of intervention
Green et al, 2015 (39)	Sample Size: N=54 Age at enrollment: 7-10 months (mean age 9 months) M:F=29:25	Efficacy study	Siblings of autistic probands (BASIS prospective longitudinal observation)	Inclusion criteria: -7-10 months baseline -siblings Exclusion criteria: -any medical disorder -being a twin -prematurity <34weeks -birthweight < 2,27 Kg	Time 1 (mean age 9 months): -AOSI -MACI -VABS II -MCDI -MSEL -GAP-Overlap -ERP	**iBASIS-VIPP intervention group (n=28):** works with parents using video-feedback to help them understand and adapt to their infant’s individual communication style to promote the best possible social and communicative development. **No intervention group (n= 26)** Duration: 5 months	Time 2 (12-15 months): -AOSI -MACI -MSEL -VABS II -MCDI -GAP overlap task (visual attention) -ERP (event-related potential to speech sounds)	-Reduced autism risk behaviors -Increased parental non directiveness -Improved attention disengagement -Improved parent-rated infant adaptive function
Green et al, 2017 (32)	Sample Size: N=54 Age at enrollment: 7-10 months (mean age 9 months) M:F=29:25	Efficacy Study Follow up at 3 years Green 2015 (27-39 months)	Siblings of autistic probands (BASIS prospective longitudinal observation)	Inclusion criteria: -7-10 months baseline -siblings Exclusion criteria: -any medical disorder -being a twin -prematurity <34 weeks -birthweight < 2,27 Kg	Time 1 (mean age 9 months): -MACI -AOSI -VABS II -MCDI -MSEL -GAP overlap task (visual attention) -ERP event-related potential to speech sounds Time 2 (12-15 months): -AOSI -MACI -MSEL -VABS II -MCDI -GAP -ERP	**iBASIS-VIPP intervention group (n=28):** works with parents using video-feedback to help them understand and adapt to their infant’s individual communication style to promote the best possible social and communicative development. **No intervention** **(n= 26)** Duration: 5 months	Time 3 (27-39 months post enrollment): ADOS2 MSEL VABS II MACI (27 months) DCMA	-Reduced severity of subsequent prodromal autism symptoms over the period to 27- 39‐month follow‐up, as well as producing positive impact on dyadic parent–infant interactions. -No significant effect on formal language or categorical ASD diagnostic outcome at 3 years
Jones et al, 2017 (34)	Sample Size N=33 Age at enrollment 6 months Age range at intervention 9-11 months M:F=21:12	Efficacy Study	High-risk infant siblings	Inclusion criteria: Older sibling with ASD Exclusion criteria: -physical signs -know genetic syndromes -serious medical or neurological conditions -neurocutaneous markings -Sensory impairment -Serious motor impairment -birthweight <2000 g -gestational age <37 weeks -History of intraventricular hemorrhage -Exposure to neurotoxins -maternal gestational diabetes -variables that may impact family functioning	Time 1 (6 months): -EEG -MSEL	**PFR intervention group (=19)**: the type of intervention focuses on promoting parenting responsivity to infant social communicative cues and behaviors using strength-based consultation strategies. **Control group (n=14):** received assessment and monitoring only Two Low-Risk comparison groups (“normative controls”) were enrolled to confirm normative patterns of responding on each task Duration: 10 weeks	Time 2 (12-18 months): -Habituation to face and objects (measure sustained attention and learning speed) -EEG theta power to social and nonsocial video (index of attention engagement) -Event-related potential (ERPs) to face and objects (Index of speed and depth of processing)	-Infants who received the intervention showed improvements in neurocognitive metrics of social attention, as reflected in a greater reduction in habituation times to face versus object stimuli between 6 and 12 months, maintained at 18 months. a greater increase in frontal EEG theta power between 6 and 12 months; and a more comparable P400 response to faces and objects at 12 months. -The high-risk infants who received the intervention showed a pattern of responses that appeared closer to the normative responses of two groups of age-matched low-risk control participants.
Watson et al, 2017 (33)	Sample Size N=87 Mean age at pretest: 14 months M:F=60:17	Efficacy Study	FYI mailing at 12 months	Inclusion criteria: -FYI high risk for a later diagnosis of ASD - birth weight >2500 g -availability of one caregiver to participate in home-based intervention sessions -primarily English spoken in the home -Non response to three contact attempts was considered a refusal of further participation. Exclusion criteria: N/A	Time 1: -FYI -CSBS -SPA -SEQ -ECBQ -MSEL -MCDI -VABS II -SRS-NV -AOSI -ADOS Parent outcomes: -PRCS -MBRS	**ART intervention group (n=45):** Adapted Responsive Teaching **REIM control group (n=42):** no direct intervention services from the research team; however, for both groups, the project coordinator contacted parents every 5 to 6 weeks for the check-in interview. Duration: 6-9 months	Time 2 (9 months post baseline, around 22 months): - CSBS - SPA - SEQ - ECBQ - MSEL - MCDI - VABS -ADOS - SRS-NV -PRCS -MBRS	-ART group parents showed significantly greater increases in responsiveness to their infants than control group parents -Changes in parent responsiveness mediated effects on child cognitive composite scores and language comprehension scores
Whitehouse et al, 2019 (26)	Sample Size N=103 Age at enrollment: 9-14 months (mean age 12 months) M:F=70:33	Efficacy Study	-Infants were recruited through the metropolitan government service for children with developmental delays, to which children are typically referred to a health professional or by a parent or caregiver self-referral -SACS-R ≥3	Inclusion criteria: -SACS-R ≥3 -age range: 9-14 months -caregiver spoke sufficient English Exclusion criteria: -diagnosed comorbidity know to affect infant neurological and developmental abilities -birth <32 weeks’ gestation -family did not intend to remain in the local area for the trial duration	Time 1 (9-14 months): -SACS-R -AOSI -MACI -MSEL -VABS II -MCDI -PSOC	**iBASIS-VIPP intervention group (N=49)** **Control group (N=48)** treatment as usual group Duration: 5 months	Time 2 (18 months): -AOSI -MACI -MSEL -VABS II -MCDI -PSOC	-A pre-emptive intervention for the autism spectrum disorder prodrome had no immediate treatment effect on early autism spectrum disorder symptoms, the quality of parent–child interactions, or researcher-administered measures of developmental skills. -Positive effect on parent-rated infant communication skills was found
Yoder et al, 2020 (40)	Sample size N=97 Age at enrollment: 12-18 months, (mean age 14 months) Male: 55%	Efficacy	-Younger sibling -Age between 12-18 months	Inclusion Criteria -younger siblings between 12-18 months at study entry -at least one full sibling with diagnosed ASD in the home -English as the primary home language Exclusion criteria: No explicit exclusion criteria	Time 1 (12-18 months): -SSIS -ALIT -CSBS -BOSCC -DPA -MacArthur -MSEL	**Improving Parents a Communication Teachers (ImPACT) group (N=49):** the curriculum focuses on setting up joint-action routines around objects that enable modeling and direct teaching of play, communication, and language skills that are just beyond the child’s current production level. **Control Group (n=48):** families assigned to the control group were free to pursue intervention outside of the research study. Parents reported their children received an average of one half-hour of non-project therapy per month Duration: 3 months	Time 2 (end of treatment): -PCFP -PCS - Specific measure: Use of ImPACT strategies Time 3 (post 6 months post-randomization): -SSIS -ALIT -CSBS -BOSCC -DPA -MCDI -MSEL Time 4 (post 9 months post-randomization): -CSBS -MCDI -MSEL -ADOS2	-Parents’ participation in ImPACT training would indirectly attenuate younger siblings’ social communication challenges 9 months later by sequentially increasing parent’s use of ImPACT strategies immediately after the intervention phase and by improving children’s midpoint proximal skills. -The ImPACT intervention had a significant effect on parents’ use of strategies, which in turn improved motor imitation and intentional communication in children.
Davis et al, 2022 (35)	Sample Size N= 87 Mean age at pretest: 14 months M:F=60:17	Efficacy Study Secondary analysis of Watson 2017	FYI mailing 12 months	Inclusion criteria: -FYI high risk for a later diagnosis of ASD - birth weight >2500 g - availability of one caregiver to participate in home-based intervention sessions -primarily English spoken in the home Exclusion criteria: - No explicit exclusion criteria	Time 1: Parent outcomes: -PRCS -MBRS	**ART group (N=45):** Adapted Responsive Teaching Intervention group **REIM control group (N=42):** no direct intervention services from the research team; however, for both groups, the project coordinator contacted parents every 5 to 6 weeks for the check-in interview. Duration: 6-9 months	Time 2 (follow up 23 months) (N=83): - MSEL **-** SC BOSCC - PRCS - MBRS	-Caregivers who participated in parent-mediated intervention improved in three domains of Caregiver Responsiveness (CR), (Contingent Verbal Sensitivity, Responsivity, Affect) -CR was also found to be a mechanism through which children’s SC skills improved. This work provides evidence that qualities of CR serve as mechanisms through which to improve and monitor child behaviors also during intervention
Whitehouse et al, 2021 (3)	Sample Size N=103. 89 participants were reassessed at age of 3 years Age at enrollment: 9-14 months (mean age 12 months) M:F=N/A	Efficacy study Follow up study of Whitehouse et al., 2019	Infants were recruited through the metropolitan government service for children with developmental delays, to which children are typically referred by a health professional or by a parent or caregiver self-referral -SACS-R ≥3	Inclusion criteria: -SACS-R ≥3 -infant was aged 9-14 months -caregiver spoke sufficient English Exclusion criteria: -diagnosed comorbidity know to affect infant neurological and developmental abilities -birth <32 weeks’ gestation -family did not intend to remain in the local area for the trial duration	Time 1 (9-14 months): -SACS-R -AOSI -MACI -MSEL -VABS II -MCDI -PSOC	**iBASIS-VIPP intervention group (N=49)** **Control group (N= 48)** treatment as usual group Duration: 5 months	Time 2 (12 months after baseline; age 24 months): -ADOS2 -MACI -MSEL -VABS II -MCDI -PSOC Time 3 (24-month postbaseline assessments; age 3 years): ADOS2 MACI MSEL VABS II MacArthur PSOC	-Receipt of a preemptive intervention for ASD from age 9 months among a sample of infants showing early signs of ASD led to reduced ASD symptom severity across early childhood and reduced the odds of an ASD diagnosis at age 3 years. -Early signs of ASD led to a small but enduring reduction in ASD symptom severity and reduced odds of ASD diagnosis in early childhood.
Bedford et al, 2024 (36)	Sample Size N=54 Age at enrollment: 7-10 months (mean age 9 months) M:F=29:25	Efficacy Study Secondary Analysis of Green et al, 2015	Siblings of autistic probands	Inclusion criteria: -7-10 months baseline -siblings Exclusion criteria: -any medical disorder -being a twin -prematurity < 34 weeks -birthweight < 2,27 Kg	Time 1 (mean age 9 months) -GAP overlap task (visual attention) -ERP (event-related potential to speech sounds -MSEL	**iBASIS-VIPP** **intervention group (n=28):** works with parents using video-feedback to help them to understand and adapt to their infant’s individual communication style to promote the best possible social and communicative development. **No intervention group (n= 26)** Duration: 5 months	Time 2 (15 months): -ERP (event-related potential to speech sounds -MSEL -Eye-tracking technology	-iBASIS-VIPP intervention resulted associated with reduced dwell time to the referent of another person’s gaze at endpoint.
Gulsrud et al, 2024 (37)	Sample Size N= 80 Age at enrollment: 12-22 months (mean age 18,21 months) M:F=64:16	Efficacy Study	-Early concerns about autism -Elevated scores on the ADOS-T	Inclusion criteria: -between 12-21 months -early concerns for autism by elevated scores on the ADOS -parent was available for session Exclusion criteria: -uncontrolled seizure activity - any known co-occurring syndromes or medical conditions	Time 1 (mean age 18,21 months): -ADOS-T - MSEL - ESCS: - SPA: Structured Play Assessment - CCX:	**Baby JASPER Intervention group (n=40):** parent-mediated Joint Attention Symbolic Play Engagement and Regulation, into a program using a standard early childhood model based on the Assessment, Evaluation and Programming System (AEPS) Curriculum for Birth to Three Years. JASPER focuses on sustaining periods of joint engagement to facilitate the development of social communication and play skills. **Control group (n=40):** infant classroom utilizing AEPS without embedded JASPER sessions Duration: 8 weeks	Time 2 (months after baseline): - MSEL - ESCS - SPA - CCX: Structured Play Assessment Time 3 (evaluation after 8 weeks): - CCX -ESCS - SPA: Structured Play Assessment	-The baby JASPER group presented increase in child initiated joint engagement and play level during dyadic interactions with their parents -Standard Baby classroom increased in joint attention during a standardized assessment delivered by an independent assessor -Infant familial risk for autism (older sibling with autism) also moderated the effect of treatment on child initiated joint engagement

Acronyms: Autistic symptoms: ESCS, Early Social Communication Scales; ADOS 2, Autism Diagnostic Observation Scale 2; AOSI, The Autism Observation Scale for Infants; SACS-R, The Social Attention and Communication Surveillance; SRS-NV, Social Responsiveness Scale-Non Verbal; BOSCC, Brief Observation of Social Communication Change. Developmental abilities: MSEL, Mullen Scales of Early Learning; Adaptive abilities: VABS, Vineland II Adaptive Behavior Scales; Communication Abilities: VABS, Vineland II Adaptive Behavior Scales (Communication subscale); CSBS, Communication and Symbolic Behavior Scale; MCDI, MacArthur Bates Communicative Development Inventory; Emotional Behavior: ITSEA, Infant-Toddler Social Emotional Assessment; ECBQ, Early childhood behavior questionnaire: short form. Sensory profile: SPA, sensory Processing Assessment for young Children; SEQ, Sensory Experiences Questionnaire; Parent-infant measures: MACI, Manchester Assessment of Caregiver-Infant interaction; SPA, Structured Play Assessment-revised; CCX, Caregiver child play interaction; PRCS, Parent Responsiveness Coding System; MBRS, Maternal Behavior Rating Scale; PSOC, Parenting Sense of Competence interest subscale; DCMA, Dyadic Communication Measure for Autism. Electrophysiological measures: GAP overlap task (visual attention); ERP, event-related potential to speech sounds. Intervention methods: ART, Adaptive Responsive Teaching; iBASIS VIPP, i BASIS Video Interaction to Promote Positive Parenting; PFR, Promoting First Relationship. In bold are reported Type of Interventions.

#### 2.4.2 Aims of the studies

The majority of studies included were efficacy studies. Only Baranek and colleagues (38) reported an efficacy and feasibility study. Two studies report long term outcomes of intervention studies published at earlier time points with the same sample (3, 32). The studies of Bedford et al. (36) and Davis et al. (35) are based on a secondary analysis respectively of Green’s 2015 cohort (eye-tracking) (39) and Watson’s 2017 cohort (parent outcome measures) (33). Therefore, the 11 studies included in this review describe only 7 cohorts (26, 33, 34, 37–40).

#### 2.4.3 Referral

There have been four main modalities of referral across studies: i) use of questionnaire tools across general population (First Year Inventory)) (33, 35, 38); ii) younger siblings of autistic children (32, 34, 36, 40); iii) early concerns of parents/health professionals (37); iv) population screening through the SACS-R (3, 26).

#### 2.4.4 Inclusion/exclusion criteria

Three out of 11 studies did not describe exclusion criteria (33, 35, 40). The other authors excluded sensory impairments (34, 38), down syndrome (38), cerebral palsy (38), significant prematurity (32, 36, 38, 39), low birth weight (36, 38, 39); being a twin (32, 36, 39), uncontrolled seizure activity (37); known syndromes or medical/neurological condition (3, 26, 32, 34, 36, 37, 39), neurocutaneous conditions (34). It is worth noting that in 5 out of 11 studies the only inclusion criterion was the status of siblings without any additional clinical inclusion criteria (32, 34, 36, 39, 40). In the other studies (33, 35, 38), participants were invited to participate if screened with a questionnaire (e.g. FYI, First Year Inventory (42)), but were secondarily enrolled in the RCT in Baranek et al. (38) if positive at the baseline assessment based on: AOSI (Autism Observation Scale for Infants (43), MSEL (Mullen Scales for Early Learning (44)), CSBS (Communication and Symbolic Behavior Scale (45),), MCDI (MacArthur Bates Communicative Development Inventory (46)), ITSEA (Infant-Toddler Social Emotional Assessment (47)), SPA (Sensory Processing Assessment for young Children (48)), SEQ (Sensory Experiences Questionnaire (49)). In Whitehouse et al. (3) infants were enrolled from well visits if they screened positive on the SACS-R (The Social Attention and Communication Surveillance- R (50)) (score>= 3). In Gulsrud et al. (2024) (37) participants were referred to the study based on parents and/or professionals’ concerns and enrolled if they met ADOS-2 Toddler Module (Autism Diagnostic Observation Scale 2, Toddler module (43)) criteria.

#### 2.4.5 Characteristics of participants at baseline

The age ranges between 7 months (26) and 22 months (37). Methods of evaluation of autistic signs at baseline comprise the AOSI (3, 26, 32, 33, 35, 36, 38, 39), the ADOS-2 Toddler Module (33, 35, 37, 38) and the SACS-R (3, 26).

#### 2.4.6 Type of outcome measures

The ADOS-2 has been the most common autism outcome measure used (3, 32, 33, 37, 38, 40), while the AOSI was used in Whitehouse et al. (3), ESCS (Early Social Communication Scales (51)) in Gulsrud et al., 2024 (37) and SRS-NV (social responsiveness scale-nonverbal (52)) in Watson et al., 2017 (33). Early social-communication competencies (including play and imitation abilities) have been measured through the SPA (Structured Play Assessment-revised (37, 53), DPA (Development Play Assessment (54)), BOSCC (Brief Observation of Social Communication Scale (55)) (40). The majority of studies investigated parental outcome through MBRS (Maternal Behavior Rating Scale (38, 56); MACI (Manchester Assessment of Caregiver-Infant interaction (57),); DCMA (Dyadic Communication Measure for Autism (32, 36, 39, 58); CCX (Caregiver child play interaction) (37), PRCS (Parent Responsiveness Coding System),MBRS (Maternal behavior Responsiveness Scale) (33, 35), PSOC (Parenting Sense of Competence (PSOC) interest subscale), MACI (Manchester Assessment of Caregiver-Infant interaction (3, 26). Communication abilities have been evaluated as outcome measures through CSBS) (32, 38, 39). Developmental outcome measures have been obtained in all studies through the MSEL scales, which assess early developmental abilities across Visual Reception, Fine Motor, Receptive Language, and Expressive Language domains (44), while the adaptive outcomes in 6 out of 11 studies (3, 26, 32, 33, 38, 39) have been obtained through VABS-2 which investigate functioning in Communication, Daily Living Skills, Socialization, and Motor Skills (59)(Sparrow, S. S., Cicchetti, D. V., & Balla, D. A (2005). Vineland Adaptive Behavior Scales, Second Edition (Vineland-II) Manual. Circle Pines, MN: American Guidance Service.).

Other clinical features have been investigated, including emotional behavior through the ITSEA (Infant-Toddler Social Emotional Assessment) (38), sensory characteristics through the SPA and the SEQ (33, 35, 38), and temperamental reactivity through the ECBQ (Early Childhood Behavior Questionnaire: short form) (33, 35). Physiological techniques, including electroencephalography (34), and eye-tracking (36), were used as objective outcome measures. Eye-tracking assessed social attention through (a) habituation time to faces vs objects (36), expecting a more reduced time for facial vs objects stimuli after intervention (tested before, after treatment and at long-term follow-up) and (b) gaze-following or dwell time (34, 39) expected to increase after intervention (measured once, after treatment, at 15 months). EEG/ERP measures included (a) expected increased frontal theta (4–6 Hz) spectral power for social vs non-social videos post-intervention (tested before, after treatment and at long-term follow-up (36)); b) evoked activity (ERP: event-related potential): visual ERPs for faces vs objects pictures - amplitude and latency of P400, expecting stronger amplitude and longer latency for faces than objects after intervention (tested before, after treatment and at long-term follow-up, 30); acustic ERPs to vowel change in speech sounds after the intervention (15 months), expecting stronger responses for changes in speech sounds after intervention (measured once, after treatment, at 15 months (34, 39)).

#### 2.4.7 Type of parent-mediated intervention

Different parent-mediated intervention methodologies have been applied across the studies:

- ART (adapted responsive teaching) (33, 35, 38). The ART (adapted responsive teaching) method consists on a 6-month, relationship-focused, home-based intervention designed to enhance parental responsiveness and child developmental outcomes. The program employs modeling and coaching to teach parents responsive interaction strategies (e.g., following the child’s lead, imitation, turn-taking) during daily routines, targeting pivotal behaviors such as social play, joint attention, engagement, and adaptability that support later development ciated with positive child outcome.
- iBasis_VIPP (video feedback intervention to promote positive parenting) (3, 26, 32, 36, 39): The manualized iBASIS-VIPP intervention consists of up to 12 individual sessions delivered in the family home by a trained therapist over five months. The primary caregiver attended all sessions, which involved videotaped parent-infant interactions used for video feedback discussions. The intervention targeted enhancement of communicative aspects of the parent-infant dyad through positive video examples, therapist-guided self-reflection, and behavioral change strategies. Parents were instructed to practice daily with their infant using the learned skills. Therapist fidelity to the protocol was monitored, and all sessions were videotaped and assessed for adherence.
- Baby Jasper (Parent mediated Joint Attention Symbolic play engagement and regulation (37). The Baby JASPER classroom-embedded sessions are group-based early interventions for infants and toddlers with early signs of autism, delivered in classroom settings and based on the Joint Attention Symbolic Play Engagement and Regulation (JASPER) model. The program targets joint engagement to support the development of social communication and play skills. Each session includes a parent-mediated component, where parents receive hands-on coaching to use JASPER strategies with their child, and an interventionist-led component, where therapists model techniques while parents observe.
- PFR intervention (Promoting First Relationship) (34): The PFR intervention promotes caregiver responsivity to infants’ social-communicative cues through strength-based, relationship-focused consultation. Caregiver–infant interactions were videotaped and reviewed to highlight parenting strengths and support interpretation of infant cues.
- ImPACT (Improving Parents as Communication Teachers) (40). The ImPACT curriculum includes strategies to promote child engagement, such as following the child’s lead, using positive affect, modeling and expanding communication, and combining interactive with direct teaching methods. It emphasizes joint action routines that support the teaching of play, communication, and language skills slightly beyond the child’s current level, with imitation of adult models encouraged. Parents were instructed to deliver at least one hour of intervention per day, five days per week, throughout the study.

## 3 Results

The current review analyzed RCT studies focused on infants and toddlers at elevated likelihood for ASD (e.g. siblings) (32, 34, 36, 39, 40) and/or presenting ASD prodromes (3, 26, 33, 35, 37, 38).

**Figure 2:**
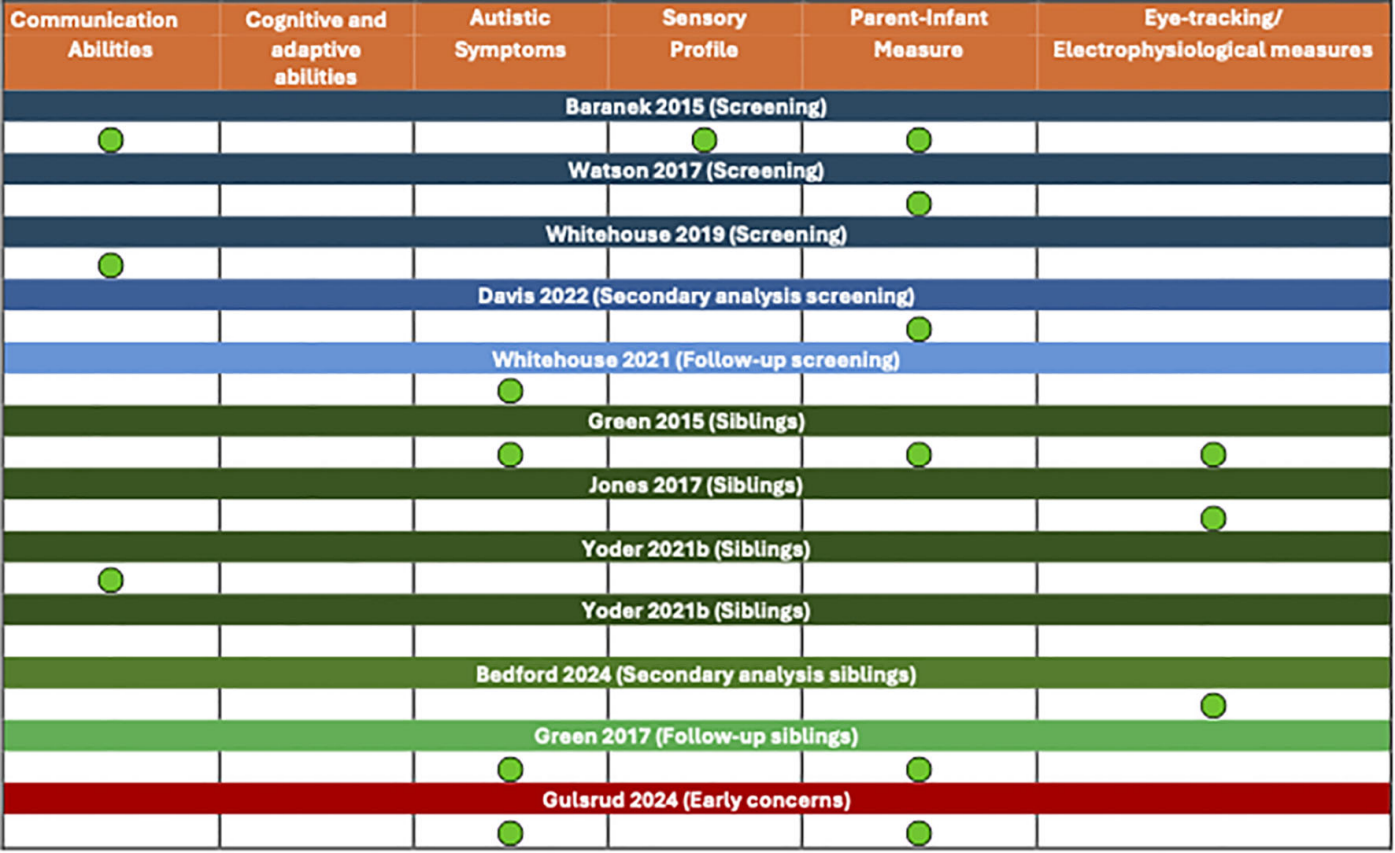
Graphical representation of results, across different development areas.

The majority of studies have reported a general efficacy of the proposed RCT program, even though expressed across heterogeneous outcome measures. It is of note that 5/11 studies compared parent-mediated intervention group with treatment as usual (3, 26, 37, 38, 40) while 3/11 compared parent mediation intervention with no intervention group (32, 36, 39). In the remaining 3 studies (33–35) the parent-mediated intervention group was compared with a group receiving only a monitoring with different timing. Specifically, 3/11 reported significant improvements on communication abilities (26, 38, 40). No study reported improvements in developmental or adaptive skills. Four studies (3, 32, 37, 39) have reported reduction of autistic symptoms in the experimental group. Two of them were based on early concerns (3, 37) and the remaining two on the same cohort from Green et al. (32, 39). Baranek et al., 2015 (38) reported significant differences as far as sensory profile impact on subjects of experimental group. Parent-infant measures resulted significantly different in the experimental group in 6/11 studies (32, 33, 35, 37–39). As far as electrophysiological measure is concerned, changes have been found in 3 papers (34, 36, 39). See **Figure 2** for a graphic representation of studies’ results.

Significant Results of RCT studies (in terms of significant differences between parent mediated intervention group and control grouped) are reported below divided into dimensional areas of development.

### 3.1 Language/communication abilities

All studies explored language/communication abilities through the following tools: a) MSEL was used in all 11 studies included in the review (3, 26, 32–40); b) VABS was used in 6 out of 11 studies included in the review (3, 26, 32, 33, 38, 39) c) MCDI was used in 7 out of 11 (3, 26, 32, 33, 38–40). Four out of 11 studies have reported significant improvements in communication skills, specifically in 2 out of 6 in terms of VABS communication (26, 38) in terms of MSEL language in one (40) out of 11 and, in terms of MCDI assessment in one (26) out of 7 studies.

### 3.2 Cognitive and adaptive abilities

All 11 studies included have used MSEL as an outcome measure while 6 studies (3, 26, 32, 33, 38, 39) have applied VABS as an outcome measure. None of the studies reported significant results in these areas.

### 3.3 Autistic symptoms

Nine out of 11 studies have explored autistic symptoms through the following measures: a) ESCS (37) b) SRS-NV (33), c) AOSI (26, 39) d) ADOS-2 (3, 26, 32, 33, 38, 39) e) VABS socialization (3, 26, 32, 33, 38, 39), f) BOSCC (35, 40).

Fourout of 9 studies reported a reduction of autistic symptoms, specifically the only study investigating symptoms with ESCS (37), in one out of two studies using AOSI (39), in two out of 6 studies applying ADOS-2 (3, 32) in one out of 6 studies using VABS socialization (39). No significant results were reported at the BOSCC assessment.

### 3.4 Sensory profile

Three studies have explored sensory profile characteristics in terms of SEQ assessment (33, 38) and SPA (33, 37, 38). No significant results were reported through SPA, while improvements in SEQ measures were reported in only one out of three studies (38).

### 3.5 Parent-infant measure

Eight out of 11 studies have explored caregiver-infant interaction through the subsequent tools: a) MBRS (33, 35, 38), b) MACI (3, 26, 32, 39), c) SPA (37), d) CCX (37); e) PRCS (33, 35), f) PSOC (3, 26). Six out of eight studies reported significant improvements in three studies of three using MBRS (33, 35, 38), in two of four using MACI (32, 39), in the only study using CCX and SPA (37), in two of two studies using PRCS (33, 35). No significant results emerged through the PSOC assessment.

### 3.6 Neurophysiological measures (eye-tracking/electrophysiological tasks)

Eye-tracking tasks: (a) Habituation time (34) was reduced to faces vs objects in the intervention group at 12 months, maintained at 18; (b) Gaze-following or dwell time (36, 39) was reduced in intervention group vs non-intervention at 15 months.

Electrophysiological measures: (a) EEG spectral power in frontal theta band (4–6 Hz) increased significantly in the intervention group for both social and non-social stimuli at 12 months, not sustained at 18; (b) Event-related potentials (ERPs): - Visual ERPs for faces and objects pictures (34): intervention groups showed larger amplitude and longer latency in P400 to faces but also to objects at 12 months; only longer latency at 18. Auditory ERPs to vowel changes in speech sounds (39): no group differences after intervention.

## 4 Discussion

The current review analyzed RCT studies focused on infants and toddlers at elevated likelihood for ASD and/or presenting ASD prodromes. Conducting RCTs focused on pre-emptive interventions for ASD prodromes is of utmost importance to establish efficacy and subsequently perhaps improve health policies. The reviewed RCTs have consistently shown the impact of pre-emptive intervention on language and intrafamilial relationships, while evidence for autistic symptom reduction is mixed. This can be attributed to the fact that only 7 cohorts (26, 33, 34, 37–40) have been studied so far, the sample sizes of the studies available are small and only 4 out of 11 studies reported a long term follow-up re-assessment (34, 37, 38, 40). It is of note that Whitehouse and colleagues (3, 26), who report the widest sample size (n=103) and re-assessment by the age of 24 months, have been able to best detect changes after treatment in communication skills (in terms of VABS communication, MCDI receptive and expressive language) at the first time point (18 months), as well as in autistic symptoms (AOSI and ADOS-2 scores) at the second time point (24 months). Furthermore the second work by the Whitehouse group (3) clearly reports a reduced probability of ASD classification in the treated groups at 36 months of age. Another possible explanation for the absence of treatment effects on core ASD symptoms is that the initial behavioral profiles or the presence of additional risk factors of participants (i.e. sex or number of ASD siblings) serve as moderators influencing treatment outcomes. Consistent with this hypothesis, Hampton et al. (27) reported that certain subgroups—such as females or children with lower cumul—may experience a reduction in the observable autistic symptoms or a delay in clinical onset following early intervention. Thus, the absence of significant average effects in group-level analyses might obscure meaningful improvements occurring within specific subpopulations. Interestingly, the second paper from Yoder’s group (60) examined pre-intervention characteristics that might describe for whom a parent-implemented intervention works. In siblings with no additional risk factors (i.e. girls with only one older sibling with autism spectrum disorder and who score low an autism spectrum disorder screen), parental receipt of “Improving Parents As Communication Teachers” training had indirect effects on children’s expressive language ability or autism spectrum disorder diagnosis through earlier effects on siblings’ intentional communication or expressive vocabulary. “Improving Parents As Communication Teachers” intervention did not show moderated or total effects on parenting-related stress or parenting efficacy. Moreover, several studies suggested that intervention benefits initially manifest in proximal behavioral domains, such as motor imitation and communicative initiations, before translating into broader improvements in core autistic symptoms (40). Additionally, enhancements in parental implementation fidelity—documented across multiple trials—are necessary but insufficient conditions for sustained symptom change (61) improving parent-mediated strategies facilitates child progress in foundational skills, yet may require complementary approaches or longer durations to have a comprehensive impact on the core symptomatology. Furthermore, children at high likelihood for autism can present an heterogeneous clinical picture in terms of symptom profiles, learning styles, and developmental trajectories (62): therefore, it is important to keep in mind possible necessity of more personalized approaches, starting from research studies that can be translated into clinical practice. In the analysis of the selected articles, it is important to separate RCTs involving the sibling population from RCTs focused on the general population presenting early ASD signs. Indeed, it is well known that siblings are more prone to develop behavioral atypicalities, and around 20% of them satisfy ASD criteria (63). Of note, the percentage of familial recurrence increases if infant gender is male, proband gender is female, and >1 older affected siblings are present (64, 65). Green et al. (2015) (39) enrolled 54 siblings in the RCT study: 28 infants (mean age 9 months) followed the iBASIS-VIPP (Video Interaction to Promote Positive Parenting) program, whereas 26 siblings did not undergo any type of treatment. The results highlighted a reduced autistic behavior, confirmed around the third year of age through the follow-up (32). The ImPACT (Improving Parents as Communication Teachers) program was indeed delivered to 47 siblings enrolled vs 48 siblings following treatment as usual outside of the research study (40). ImPACT intervention had a significant effect on parent’s use of strategies, which in turn improved motor imitation and intentional communication in children allocated to the intervention arm.

The articles included in the current review which are not focused exclusively on a siblings population, can be divided into: 1) studies where screening questionnaires have been used as referral tools, and subsequently the clinical assessment was delivered in order to select children to be included in the RCT (33, 35, 38); 2) studies where children were referred due to early concerns of parents/health professionals (34); 3) studies where an observational screening tool, e.g. SACS-R, was applied to the general population in order to enroll children who failed the screening (3, 26).

The three studies which used screening questionnaires, all used the FYI tool (42) sending it by mail as a screening questionnaire; the instrument can be applied starting from 12 months of age and is self-compiled by parents. Its feasibility as a screening tool has been evaluated in recent decades (66)., also with machine learning approaches (67) but it is not mentioned as gold standard in a recent review by Salgado Cacho (68). As FYI is a questionnaire filled out by caregivers, all studies added clinical assessment after screening, thus making it possible to catch detailed changes in tools used after treatment. However, FYI is a self-compiled questionnaire and some higher likelihood for autism infants can easily be missed. It is of interest that SACS-R (50), an observational tool administered by health professionals, has been used by the Whitehouse group (3, 26) as a measure to detect early autistic signs and subsequently enroll infants in a RCT study. At baseline the authors also administered AOSI to measure autistic signs. Gulsrud et al. (37), enrolled 80 toddlers referred due to parental concerns, and confirmed the presence of ASD signs at the T0 assessment through the administration of ADOS-2 Toddler Module.

Few RCTs in this systematic review employed neurophysiological or visual-behavioral measures to assess intervention impact on social brain networks. Although these approaches provide valuable insights into early social development and potential ASD biomarkers, methodological heterogeneity remains high and no specific markers have been validated, yet. Evidence in literature indicates that infants at high likelihood for autism show reduced facial sensitivity, atypical hemispheric lateralization, and altered EEG spectral power (69). Among reviewed studies, only two included neurophysiological assessments, both with small samples and low compliance for EEG/ERP tasks, while eye-tracking achieved higher retention Only Jones et al. (2017) conducted repeated post-intervention assessments, showing limited longitudinal consistency between 12 and 18 months.

Eye-tracking measures revealed significant intervention effects on social behavior. Jones et al. (34) reported a sustained reduction in habituation time to faces versus objects, while Green et al. (2015; reported by Bedford et al. (36, 39)) found reduced dwell time in joint attention tasks, contrary to expectations. Although shorter dwell time has been linked to higher autism likelihood (Bedford et al., 2012) (70), the iBASIS-VIPP intervention reduced autism-related behaviors longitudinally (32) (Green et al., 2017). In the same cohort, *Green* et al. (39) found poorer auditory ERPs, previously associated with weaker language outcomes ( (71); however later follow ups showed language improvements confirmed in replication (3, 32). These delayed benefits may reflect later-emerging language gains from enhanced early social engagement, consistent with social-interactionist theories and the “resource competition” model, suggesting early social input may transiently limit language development. This underscores the need to monitor both intended and unintended effects of early interventions (72) (Bottema-Beutel et al., 2021).

Electrophysiological results were also variable. Green et al. (2015) (39) found no intervention effects on auditory ERPs from a speech oddball task, whereas Jones et al. (2018) (34) reported electrophysiological responses to visual stimuli sensitive to treatment. In the latter, EEG theta-band (4–6 Hz) activity during social and non-social video viewing and ERPs to faces versus objects were examined as indicators of neural specialization for social processing. The intervention group showed greater theta power at 12 months, though not specific to social stimuli, and effects diminished by 18 months, possibly reflecting weaker responses to video versus live stimuli (73) (Jones, 2015). Similarly, P400 measures showed enhanced responses to faces shortly after treatment but weaker effects later. Since, reduced P400 responses are linked to early autistic behavior (69, 73, 74) (Clairmont, 2021; Jones, 2016; Elsabbagh, 2012), this suggests that early interventions can transiently influence neural mechanisms of social processing. The attenuation over time underscores the need to optimize intervention duration and include long-term clinical and electrophysiological follow-ups to assess sustained social brain changes.

All intervention proposals share the principle that the involvement of parents in the early phases of emerging symptoms is of utmost importance to empower family awareness about the condition and consequently family well-being (75). Indeed, in 9 out of 11 (3, 26, 32, 35, 37–40) studies included in the current review, caregiver-infant interaction was investigated in pre-post intervention evaluations. For example, significant reduction in MBRS directiveness was detected in the treated group in Baranek et al. (38), improvements at the MACI scale were reported in Green et al. (39) and Green et al. (32). Watson and colleagues (33) report that changes in parent responsiveness, measured by MBRS and PRCS, significantly mediated change in more than half of child outcome measures, consistently with some previous literature (61, 76), thus supporting importance of involving parents on intervention programs. On the other hand, in the work from Yoder’s group (60) ImPACT teaching did not show positive or negative effects on parents’ stress or efficacy, regardless of whether parents’ initial depressive symptoms are considered, while an indirect effect of ImPACT teaching was found on expressive abilities in children, in the subgroup with no additional risk (e.g. i.e. girls with only one older sibling with autism spectrum disorder and who score at low risk on an autism spectrum disorder screen).

More generally, early ASD intervention shows a mixed pattern of direct versus indirect effects. Direct, child‐level gains (e.g., reduced symptom severity, joint engagement, and early social‐communication) are detectable but often modest and short‐lived unless intensity and fit are high; for instance, Baby JASPER produced proximal improvements in child‐initiated joint engagement and play, while broader or longer‐term symptom change was limited without continued coaching (37). In contrast, indirect pathways—changes in parent behavior that subsequently shape child outcomes—are more consistently supported: in the ART trial, parent responsiveness mediated treatment effects on child social-communication, highlighting parent style as the mechanism of impact (33). Moreover, parental outcomes can moderate the pathway: in a multisite RCT, the effect of early intensive intervention on parents’ sense of efficacy depended on baseline stress, underscoring that optimizing caregiver well-being may be necessary to realize child benefits (77). Consistent with these findings, McGlade et al. (2023) (28) concluded that very early interventions yield low-to-moderate certainty of limited direct neurodevelopmental change by age 3, suggesting that robust parent-mediated mechanisms (rather than large immediate child-symptom shifts) may best explain early benefits.

Asfar as iBASIS-VIPP is concerned, it has been reported (78) that this kind of early intervention could represent a good-value societal investment for supporting neurodivergent children, thus implicating cost savings and suggesting that preemptive interventions may be a feasible, effective, and efficient new clinical approach in the autism field, reducing disability and the costs of support services, even though long-term follow-up of children receiving preemptive intervention is needed to confirm the modeled results.

The general positive results of the reviewed studies, despite limited and preliminary, warrant additional development of pre-emptive intervention models and additional rigorous randomized control trials. The initial findings suggest that pre-emptive intervention has the potential to support children development and family well-being, thus reducing cost of support services. In case additional studies demonstrate long-term positive impact of pre-emptive intervention the rationale for referring high likelihood infants is even more powerful.

The current work underlines the importance of investigating the efficacy of very early intervention in children at likelihood for autism. Though evidence regarding autistic symptoms reduction is still limited, wider evidence demonstrates the positive effects of early intervention in children’s developmental trajectory, in terms of communication and cognitive skills, and additionally a positive effect on parent-child relationship, and their direct (and indirect) effects on outcome profile. Another interesting topic could be involving caregivers other than parents, in order to enlarge feasibility of the intervention, as the pilot study from Dongmei Ge et al. (79) suggests.

Larger cohorts are needed to generalize emerging efficacy evidence and to develop tools increasingly able to catch developmental gains (also in terms of different autistic dimensions, e.g. socio-communicative difficulties vs repetitive behaviors) in RCT studies., in addition ethical issues need to be taken into consideration, as they are a matter of lively debate (80). Indeed, it is very important to consider the challenges inherent in treating risk (not disorder) and taking into account the tremendous heterogeneity associated with ASD and the nature of ASD-associated behaviors that can be egosyntonic (ie, compatible with an individual’s values and beliefs about themselves) (81).

It is of utmost importance to consider the research-to-practice gap (82) that is dominant at the moment, and try to reduce it as much as possible in order to make early intervention possible for all children at likelihood for autism and their families. Investing federal and philanthropic research funds in ASD service research could enhance current health systems and prepare them for the necessary expansion required to deliver presymptomatic testing and interventions. While some may regard service research connected to not-yet-validated predictive biomarkers as premature, in reality, advancing biomarker research and service research in parallel is crucial for new predictive methods to reach their clinical potential and to prevent further disadvantaging children and families who already struggle to obtain diagnostic and intervention services for ASD (81).
